# Altered Protozoan and Bacterial Communities and Survival of *Escherichia coli* O157:H7 in Monensin-Treated Wastewater from a Dairy Lagoon

**DOI:** 10.1371/journal.pone.0054782

**Published:** 2013-01-22

**Authors:** Subbarao V. Ravva, Chester Z. Sarreal, Robert E. Mandrell

**Affiliations:** Produce Safety and Microbiology Research Unit, U.S. Department of Agriculture, Agriculture Research Service, Western Regional Research Center, Albany, California, United States of America; University of Edinburgh, United Kingdom

## Abstract

Surviving predation is a fitness trait of *Escherichia coli* O157:H7 (EcO157) that provides ample time for the pathogen to be transported from reservoirs (e.g. dairies and feedlots) to farm produce grown in proximity. Ionophore dietary supplements that inhibit rumen protozoa may provide such a selective advantage for EcO157 to proliferate in lagoons as the pathogen is released along with the undigested supplement as manure washings. This study evaluated the fate of an outbreak strain of EcO157, protozoan and bacterial communities in wastewater treated with monensin. Although total protozoa and native bacteria were unaffected by monensin, the time for 90% decrease in EcO157 increased from 0.8 to 5.1 days. 18S and 16S rRNA gene sequencing of wastewater samples revealed that monensin eliminated almost all colpodean and oligohymenophorean ciliates, probably facilitating the extended survival of EcO157. Total protozoan numbers remained high in treated wastewater as monensin enriched 94% of protozoan sequences undetected with untreated wastewater. Monensin stimulated 30-fold increases in *Cyrtohymena citrina*, a spirotrichean ciliate, and also biflagellate bicosoecids and cercozoans. Sequences of gram-negative *Proteobacteria* increased from 1% to 46% with monensin, but gram-positive *Firmicutes* decreased from 93% to 46%. It is noteworthy that EcO157 numbers increased significantly (*P<*0.01) in Sonneborn medium containing monensin, probably due to monensin-inhibited growth of *Vorticella microstoma (P<*0.05*),* a ciliate isolated from wastewater. We conclude that dietary monensin inhibits ciliate protozoa that feed on EcO157. Feed supplements or other methods that enrich these protozoa in cattle manure could be a novel strategy to control the environmental dissemination of EcO157 from dairies to produce production environments.

## Introduction

Consumption of contaminated produce has been responsible for 19% of all *Escherichia coli* O157:H7 (EcO157) outbreaks during 1998 to 2007 [1]. Major outbreaks associated with produce indicate that pre-harvest contamination in the field has occurred often, so it is critical to identify sources of pathogens in the environment and interventions for minimizing them [2]. Pathogens attached to contaminated ‘ready to eat’ produce are difficult to remove [3], therefore, prevention of pre-harvest contamination is critical. Controlling pathogens in livestock and agricultural environments to minimize pre-harvest contamination will require an understanding of the biological and environmental factors that regulate the proliferation of pathogens during their transport from animal reservoirs, e.g. livestock operations and wildlife, to produce grown in proximity to point sources.

The survival and growth of EcO157 in the environment are important factors in pre-harvest contamination. EcO157 has been reported to survive as long as 21 months in manure piles exposed to fluctuating environmental conditions [4], in contrast to the 90% decline rates within 1 to 10 days in wastewater from different dairy lagoons we reported for several strains of EcO157 [5]. Environmental factors that cause fluctuations in enteric pathogens provide opportunities to alter the chemical or biological nature of the environment and minimize pre-harvest contamination of fruits and vegetables grown in proximity to dairies. The rapid decline of EcO157 populations in dairy wastewater due to inhibitory chemicals [6] and protozoan predation [7] are examples of fluctuations that could be exploited to decrease the pathogen.

Ruminants are primary reservoirs of EcO157 shedding as high as 10^7^ cells per gram of feces [8]. Eventually, the pathogen is transported as manure washings from the dairy barns to waste lagoons. Protozoa constitute half of the microbial biomass in the rumen [9], however, there have been few reports of any interactions between rumen protozoa and shiga-toxin producing *E. coli* from actively shedding cattle [10]. The protozoan predators of EcO157 in dairy wastewater we isolated and characterized [7] did not originate from cows [11], [12]. Indeed, anaerobic rumen protozoa in feces fail to survive in waste lagoons.

Protozoan predation was linked indirectly to decreases in EcO157 in drinking water wells [13] and wastewater from a dairy [6]. Direct evidence of predation by *Acanthamoeba polyphaga* of enteropathogenic *E. coli* [14] and ciliates isolated from store-bought spinach and lettuce that graze on EcO157 have been reported previously [15]. We reported recently that *Colpoda aspera* and *Platyophrya* species from dairy wastewater consumed EcO157 in preference to native aerobic bacteria [7]. In contrast, *Vorticella microstoma,* also isolated from wastewater, consumed, but failed to eliminate or digest EcO157 [7]. Similarly, *E. coli* and other pathogenic bacteria can survive and proliferate inside protozoa from the nutrients available in vacuoles [16], [17]. Thus, passage through protozoa provides the pathogenic bacteria a survival advantage by persisting in inhospitable aquatic environments such as chlorinated waters [16], but also may increase in virulence as a human pathogen by adapting to an intracellular lifestyle [18]–[20].

In addition to surviving within protozoa, pathogenic *E. coli* can be resistant to veterinary pharmaceuticals commonly used as feed additives [21]. Monensin, a polyether ionophore antibiotic, is widely used as a dietary supplement to modify ruminal microbial communities for bloat relief by reducing methane emissions and increased milk production by improving energy metabolism in dairy cows [22]. Monensin inhibits rumen ciliates [23] and gram-positive bacteria, but not gram-negative bacteria [24], [25], including EcO157 [26]. Thus, monensin has been used for selective growth of gram-negative bacteria by inhibiting other microflora [27]. It is noteworthy that the introduction of ionophores used in the diet of 90% of feedlot and farm-fed cattle roughly coincided with the identification of EcO157 as a foodborne human pathogen, and stimulating speculation that ionophores enhanced the ability of EcO157 to become established in the bovine gut [26], [28]. A tentative association between prevalence of EcO157 and the use of ionophores was also reported [29] but not substantiated.

Our previous reports of the increase of EcO157 in protozoa-free wastewater [6] and decrease in protozoa-rich [7] dairy lagoon wastewater stimulated us to identify protozoan communities responsible for the decrease and/or elimination of EcO157. We monitored the fate of an outbreak strain of EcO157 in wastewater treated with or without monensin. Protozoan and bacterial community shifts correlated directly to extended survival of EcO157 were characterized by creating 18S and 16S rRNA gene sequence libraries from DNA extracted from wastewater supplemented with or without monensin. The fluctuations in populations of resident aerobic bacteria and protozoa were monitored during the extended incubation period. In addition, the direct influence of monensin on the survival of EcO157 in the presence or absence of protozoa in Sonneborn medium was evaluated.

## Materials and Methods

### Ethics Statement

Wastewater samples were collected with permission from a private dairy located in Modesto, California [7] without unduly disturbing the cows.

### Survival of EcO157 in Wastewater Treated with Monensin

Freshly collected wastewater from a lagoon from a medium-sized dairy in central California [7] was used to determine the survival of EcO157 in the presence and absence of monensin. The chemical composition of wastewater was similar to that of the unfiltered wastewater described earlier [6]. One-hundred milliliters of unfiltered wastewater in triplicate 250 ml Erlenmeyer flasks were treated with or without 1500 µg/ml of monensin for four days (90–95%, Sigma-Aldrich, St. Louis, MO) followed by inoculation with EcO157. Pre-incubation with monensin was designed to maximize the inhibition of native protozoa. The high concentration was chosen arbitrarily as monensin was not inhibitory to *E. coli* at 2048 µg/ml [30] and it degraded rapidly in soil with a half-life of <4 d [31]. The treated wastewaters were inoculated with 6.6×10^7^ CFU/ml of EcO157 strain MM123 and incubated at 25°C for 14 days on a gyratory shaker operated at 50 rpm. Low speed mixing simulates circulating aerators used in dairy lagoons [5]. MM123 is a spontaneous rifampin-resistant (100 µg/ml) mutant of green-fluorescent-protein labeled apple juice outbreak strain RM2315 (wild-type: FDA strain SEA13B88) [5], [32]. Rifampin resistance aids in discriminating MM123 colonies during enumerations from bacteria in wastewater [6]. EcO157 grown overnight in LB broth supplemented with 100 µg/ml of rifampin and 50 µg/ml of kanamycin, was centrifuged and resuspended in 0.01 M phosphate-buffered saline (PBS; pH 7.4) and used as inoculum.

The populations of green-fluorescent-protein labeled EcO157, aerobic bacteria, and protozoa were monitored at various intervals as described previously [7]. Repeated measures 2-way ANOVA (Prism 5.0; GraphPad Software, Inc., San Diego, CA) with Bonferroni multiple comparisons was used to compare differences in the growth of EcO157, protozoa and bacteria in monensin-treated and untreated wastewaters. Bacterial and protozoan communities from monensin treated and untreated wastewater following a 14-day incubation period were characterized by 16S and 18S rRNA gene sequencing.

### Effect of Monensin on the Consumption of EcO157 by Monocultures of Protozoa

Consumption of EcO157 strain MM123 in the presence of monensin was determined using *V. microstoma* and *C. aspera* isolated previously [7] from dairy wastewater. Sterilized 10% Sonneborn medium (Solution 1 of ATCC medium 802, http://www.atcc.org/Attachments/4018.pdf) in 0.01 M PBS supplemented with or without 1500 µg/ml of monensin was inoculated with 1×10^8^ CFU/ml of EcO157 and 2×10^3^ cells of protozoa per ml (50,000 bacteria cells per protozoa cell). A 7-day growth of protozoa from full-strength Sonneborn medium was used as inoculum. Overnight growth of EcO157 strain MM123 in LB broth supplemented with 50 µg/ml of kanamycin, centrifuged and resuspended in 0.01 M PBS was used as the bacterial food source for protozoa. The comparisons were in triplicate and treatments to determine the direct influence of monensin on only protozoa or EcO157 were also included. The populations of both EcO157 and protozoa during a 6-day incubation without agitation at 25°C were determined using methods described earlier [7]. Stationary incubations aid in grazing of EcO157 by micro-vortexing and filter feeding by the ciliates. Two-way ANOVA coupled with Bonferroni post-tests was used to compare differences in growth of protozoa and EcO157 in the presence or absence of monensin.

### Extraction of DNA from Wastewater

DNA was extracted from wastewater using the MoBio UltraClean soil DNA isolation kit (MoBio, Solano Beach, CA) with some modifications of the manufacturer’s “alternate protocol” to obtain maximum yield. Brieﬂy, 50 ml of treated or untreated wastewater were centrifuged at 20,000×*g* for 10 min and the pellet (∼0.5 g) was suspended in 1 ml of sterile water and mixed with the kit-supplied beads in a 2-ml solution tube plus an equal amount of 0.1-mm zirconia-silica beads. The cells were disrupted by placing the tubes in a Mini-Beadbeater-8 (Biospec Products, Bartlesville, OK) and operating it for five 60 second pulses alternating with 60 seconds on ice. The samples were then subjected to two alternate freeze (−80°C, 5 min)/thaw (65°C, 5 min) cycles and treated with 30 µg of proteinase K for 1 h at 60°C. The remainder of the protocol was preformed as described by the manufacturer except that the DNA collected in 50 µl of UltraClean PCR water (MoBio) was re-purified twice by repeating steps 15 to 21 of the “alternate protocol.” Purified DNA that was devoid of green color was suspended to a concentration of 5 ng/µl in UltraClean PCR water.

### 16S and 18S rRNA Gene Sequence Clone Libraries and DNA Sequence Analysis

Amplification of 16S rRNA gene sequences was carried out as described by Hernlem and Ravva [33] using the eubacterium specific primers 27f (5′ AGAGTTTGATCCTGGCTCAG 3′) and 1392r (5′ GACGGGCGGTGTGTAC 3′). Creation and sequencing of 16S rRNA clone libraries was the same as described earlier [34], except that the reverse primer 1392r was used for unidirectional sequencing reactions. Methods for amplification and sequencing of 18S rRNA gene sequences using the protozoa-specific forward primer P-SSU-342 (5′-CTTTCGATGGTAGTGTATTGGACTAC-3′) and a eukarya-specific reverse primer Medlin B (5′-TGATCCTTCTGCAGGTTCACCTAC-3′) were as described previously [7].

DNA sequence analysis and dendrogram construction were done using Kodon v. 3.6 and Bionumerics v.6.5 (Applied Maths, Inc., Austin, TX). Phylogenetic trees were constructed by using a neighbor-joining method incorporating Jukes-Cantor distance correction. Tree stability was assessed by bootstrap analysis with 1,000 iterations and bootstrap values >70% are noted at each node. Taxonomic assignment of 16S rRNA sequences to the bacterial classes was done with 100% confidence level using the RDP Naïve Bayesian rRNA Classifier Version 2.2 of the Ribosomal Database Project [35]. Protozoan classification was based on the genus search on the taxonomy module of the UniProt database (http://www.uniprot.org/taxonomy/) and the nameless ranked systematics of Adl *et al*. [36]. Rarefaction curves and species coverage estimates for 18S and 16S rRNA clone libraries from each sample were generated using operational taxonomic units (OTUs) with >97% sequence similarity with GenBank accessions [34]. Species richness and diversity indices were determined for each library of OTUs using Simpson’s and Shannon-Wiener index of diversities (Bionumerics).

### Nucleotide Sequence Accession Numbers

Representative DNA sequences of all protozoa and the top 20 predominant bacterial sequences derived from wastewater treated with or without monensin were submitted to GenBank under accession numbers HQ293222 to HQ293271 and JF423913.

## Results

### Survival of EcO157 in Wastewater Treated with Monensin

**Figure 1:**
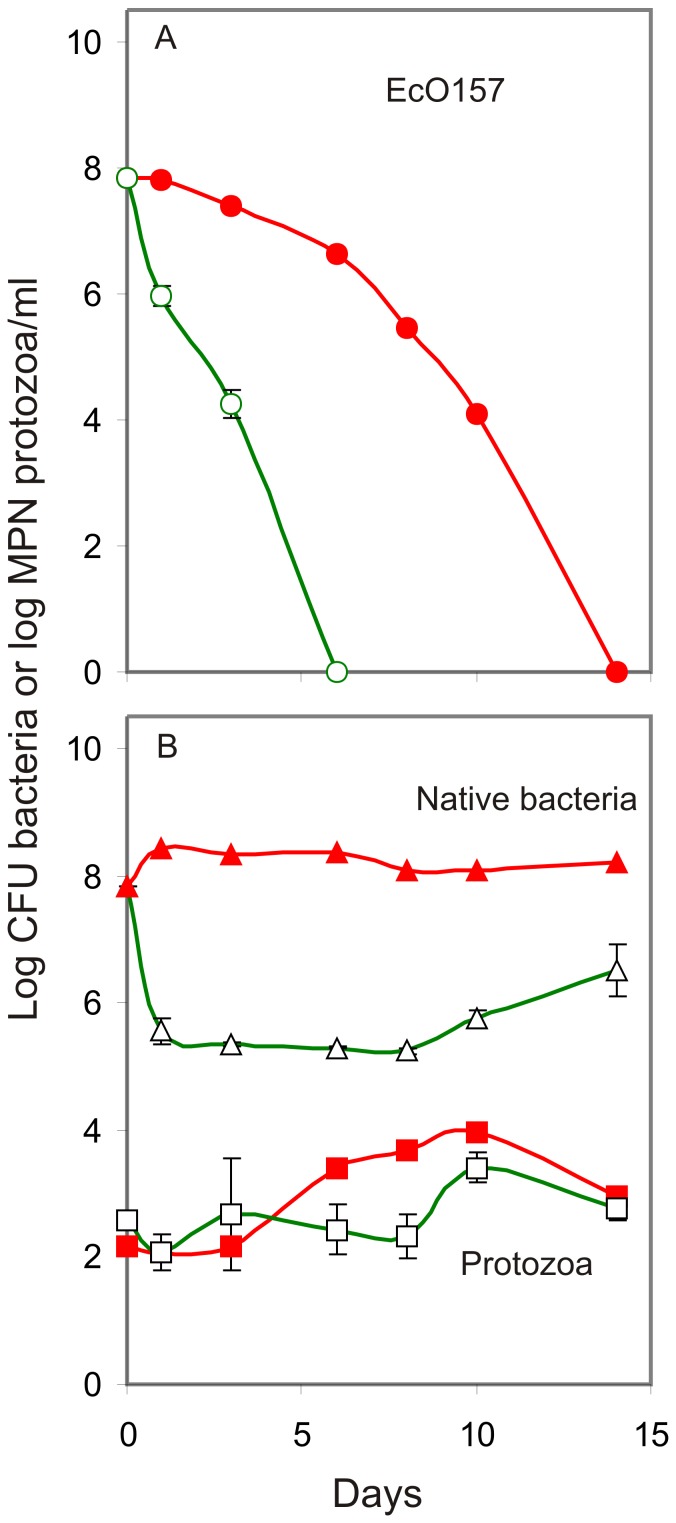
Survival of EcO157 in dairy wastewater treated with monensin. EcO157 populations (top panel) were monitored in wastewater treated with (red filled circle) or without (green open circle) monensin. Aerobic bacteria (triangles) and native protozoa (squares) in the presence (red, filled symbols) or absence (green, open symbols) of monensin were also monitored (bottom panel). The monensin-treatment replicate (panel A, red line) with extended survival of EcO157 was chosen for characterization of protozoan and bacterial communities by 18S and 16S rRNA gene sequencing. Corresponding data for total bacteria and protozoa for the replicate are plotted (bottom panel, red lines). Standard deviations for averages of triplicates for untreated wastewater are shown. Replicate differences in survival of EcO157 in monensin-treated wastewater are shown in Figure S1.

Monensin treatment of wastewater extended the survival of EcO157; 6.6 log CFU were detected after 6 days (Figure 1A), whereas, in untreated wastewater no EcO157 cells were detected. Direct inoculation in wastewater resulted in a 90% decrease (D-value) of EcO157 cells in 0.8±0.0 days, whereas, monensin treatment increased D-value to 3.5±1.4 days (Figure S1). Although replicate D-values fluctuated, EcO157 populations were significantly higher (*P*<0.0001, F = 384; Figure S1A) with monensin treatment. Furthermore, monensin treatment resulted in a significant increase (*P*<0.05, Bonferroni t_day 1_ = 3.2; *P*<0.001, F_time factor_ = 15; Figure S1A) in total bacteria that remained at elevated levels throughout the incubation, but failed to inhibit (*P* = 0.47) the native protozoan populations (Figure 1B). The monensin treatment, which resulted in prolonged viability of EcO157 (D-value, 5.1 d; Figures 1 and S1) and increased numbers (*P*<0.0001; t>_1 d_ = >6) of native bacteria, was used for characterization of protozoan and bacterial communities.

### Protozoan Communities in Untreated and Monensin-treated Wastewaters

**Figure 2:**
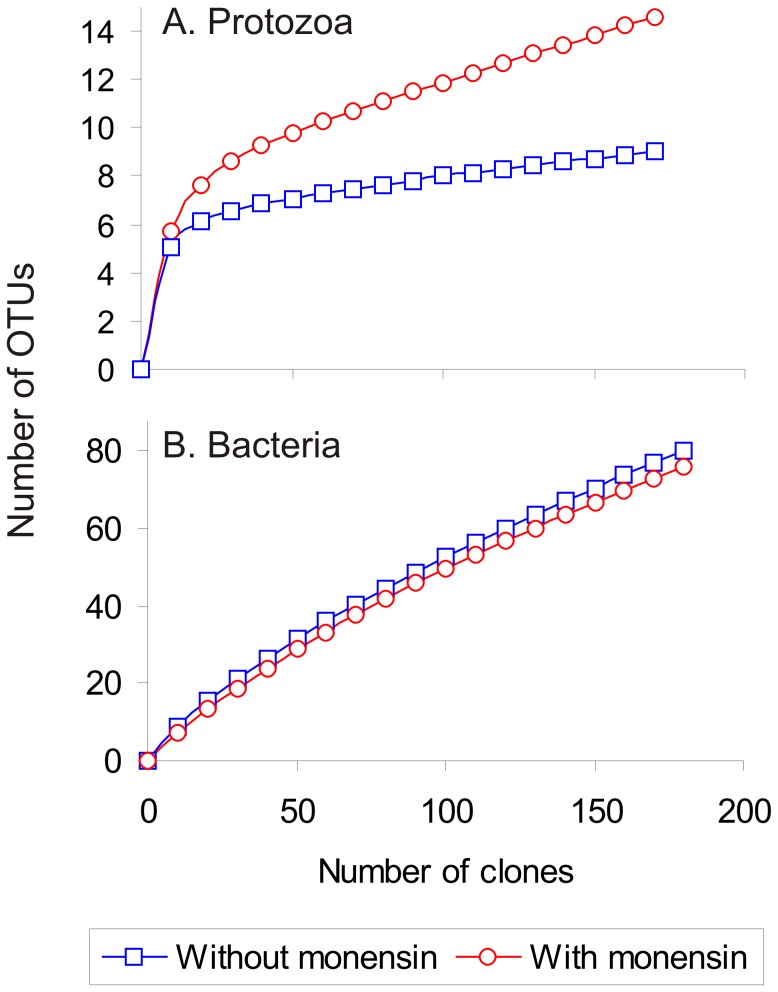
Rarefaction curves for 18S rRNA and 16S rRNA clone libraries created from DNA extracted from wastewater treated with or without monensin. Freeware program aRarefactWin (v.1.3) by Holland (http://strata.uga.edu/software/win/aRarefactWin.exe) was used for rarefaction analysis and species coverage estimates of clone libraries of protozoa (A) and bacteria (B).

**Figure 3:**
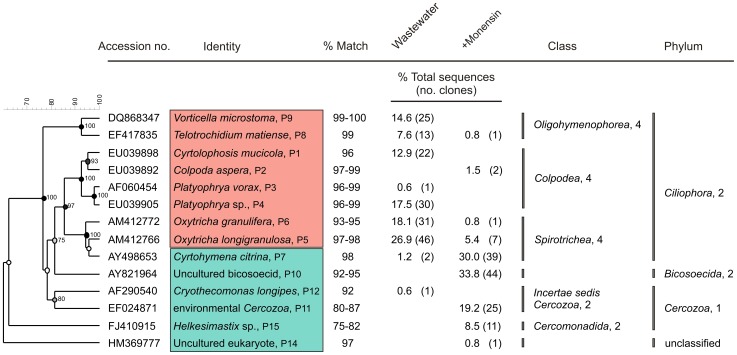
Protozoa characterized from dairy wastewater treated with or without monensin. Numbers P1 to P14 and P15 in the identity column correspond with DNA sequences submitted to GenBank under accession numbers HQ293258 to HQ293271 and JF423913. The number in parenthesis following the percentage of sequences is the number of clones that best matched with the GenBank sequences. Numbers following the traditional class and phylum designations are the ranks based on the new nameless ranked systematics of Adl *et al.* [36]. Phylum *Cercozoa* was elevated to first rank and the order *Cercomonadida* to a second rank by this classification. The identity of *Helkesimastix* sp. is based on only 75 to 82% match with the GenBank sequences and could be classified as an uncharacterized cercozoan. Red and green boxes respectively denote inhibition or enrichment of protozoa by monensin.

Of a total of 299 protozoan sequences characterized, 168 and 131 were from untreated and monensin-treated wastewater, respectively. Monensin altered the protozoan community structure by increasing the species diversity and richness as indicated by Shannon-Wiener and Simpson diversity indices and species coverage estimates (Table 1). Rarefaction analysis (Figure 2A) indicated that the protozoan species diversity is generally low in wastewaters as indicated by low species coverage estimates (5 to 8%; Table 1). Although sampling was not exhaustive, the predominant protozoa were characterized, probably, as indicated by the decrease in the slope of the curve towards the endpoint for untreated wastewater as compared to monensin treatment (Figure 2A). Members of the phylum *Ciliophora* represented 99% of all the sequences characterized from wastewater and were decreased to 39% with monensin treatment (Table 2 and Figure 3). Monensin treatment decreased the colpodean ciliate sequences from 31% to 2% and the oligohymenophorean ciliates from 22% to 1% (Table 2), in contrast to increased sequences representing *Cyrtohymena citrina,* a spirotrichean ciliate, from 1% to 30% (Figure 3). Monensin facilitated an increase in the proportion of biflagellate bicosoecid sequences to 34% and cercozoans sequences to 28% as compared to these two being nearly undetectable in untreated wastewater (Table 2). In addition, 94% of protozoan sequences that were enriched with monensin were not detected in untreated wastewater and resident members of wastewater belonging to the genus *Platyophrya* were eliminated by monensin (Figure 3). Ciliate sequences of *Cyrtolophosis mucicola* and *Vorticella microstoma* detected at high levels in wastewater were not detected after monensin treatment (Figure 3).

**Table 1:** Species diversity and richness of bacterial and protozoan communities in dairy wastewater treated with or without monensin.

Microbial communities	Monensin-treated^a^			Without monensin^a^		
	Shannon-Wiener	Simpson	Species coverage	Shannon-Wiener	Simpson	Species coverage
Protozoa	2.07	0.851	8.6%	1.82	0.825	5.3%
Bacteria	2.38	0.770	33.5%	2.93	0.928	44.5%

a OTUs with >97% sequence similarity with GenBank accessions were used in determining the diversity indices and for rarefaction analysis. Species coverage estimates are calculated from rarefaction data.

**Table 2:** Protozoa characterized from wastewater and wastewater treated with monensin.

	Percent total characterized sequences	
Protozoan class and phylum	Without monensin	Monensin-treated
*Colpodea, Ciliophora*	31	2
*Spirotrichea, Ciliophora*	46	36
*Oligohymenophorea, Ciliophora*	22	1
*Bicosoecida* a	0	34
*Cercozoa* a	1	28
Other unclassified	0	1

a Phylum.

### Bacterial Communities in Wastewater

**Figure 4:**
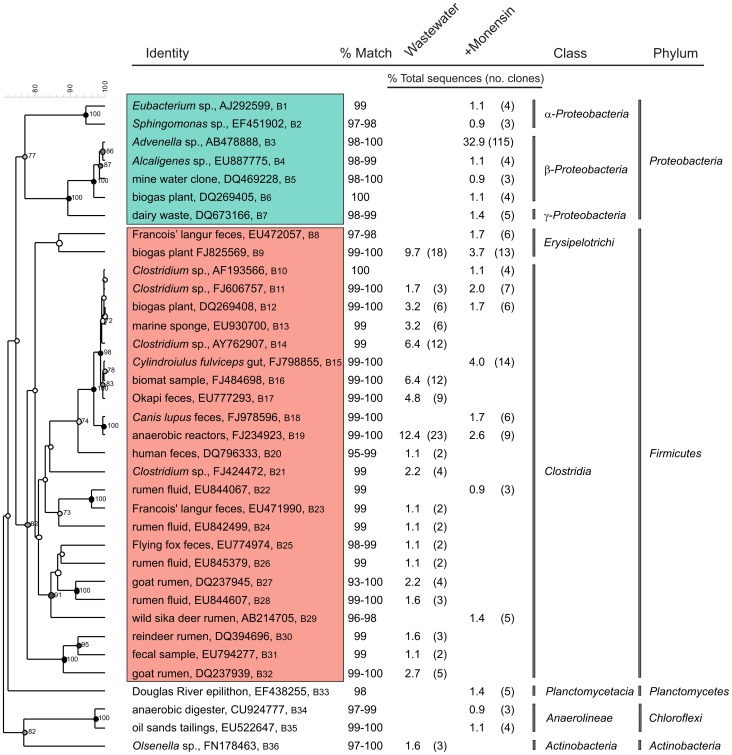
The 20 predominant bacterial sequences characterized from dairy wastewater treated with or without monensin. Numbers B1 to B36 in the identity column correspond to sequences submitted to GenBank under accession numbers HQ293222 to HQ293257. The numbers in parentheses following the percentage of total sequences are the number of clones that best matched with the GenBank sequences. Red and green boxes respectively denote inhibition or enrichment of bacteria by monensin.

16S rRNA sequence libraries of 181 and 346 clones for wastewater monensin-treated wastewater, respectively, were characterized from DNA extracted 14 days after inoculation with EcO157. A second sequence library of an equal number of cloning reactions were performed for monensin-treated wastewater to confirm the unusually high number of sequences representing *Proteobacteria* (Table 3) and the increases in number of sequences for *Advenella* species from an undetectable level to 33% (Figure 4). This finding was supported by a decrease in bacterial species diversity with monensin treatment as indicated by decreases in Shannon-Wiener and Simpson diversity indices and species coverage estimates (Table 1). Rarefaction analysis indicated that the sampling was not exhaustive enough to fully characterize the bacterial communities as indicated by the absence of a plateau towards the endpoint of the curves for both clone libraries obtained from wastewater treated with or without monensin (Figure 2B).

**Table 3:** Bacteria characterized from dairy wastewater treated with or without monensin and inoculated and incubated with EcO157 for 14 days.

	Percent total 16S rRNA sequences (no. of clones)			
Bacterial phylum	Without monensin		Monensin-treated	
	All	Top 20	All	Top 20
***Firmicutes*** a	93.4 (169)	97.5 (118)	45.7 (158)	32.7 (73)
***Proteobacteria*** a	1.1 (2)	0 (0)	46.2 (160)	61.6 (138)
*Chloroflexi*	0 (0)	0 (0)	2.9 (10)	3.5 (8)
*Actinobacteria*	5.0 (9)	2.5 (3)	2.3 (8)	0 (0)
*Planctomycetes*	0 (0)	0 (0)	1.7 (6)	2.2 (5)
*Bacteriodetes*	0.6 (1)	0 (0)	0.3 (1)	0 (0)

a Boldface type designates the phyla that resulted in a major difference in detection in samples with and without monensin.

The top 20 bacterial sequences characterized from wastewater matched the uncultured bacterial sequences from anaerobic digestors, rumen fluids and fecal samples determined previously (Figure 3). All of them were gram-positive and 97.5% of the clones representing these sequences were identified as members of *Firmicutes* (Table 3), whereas the other 2.5% belong to phylum *Actinobacteria*. Monensin treatment decreased the gram-positives to 33% of the top 20 sequences. Anaerobic bacteria in the class *Clostridia* represented 83% of the top 20 sequences (100/121 clones), but decreased to 24% (54/224 clones) with monensin treatment (Figure 4). In addition, sequences of gram-positive *Chloroflexi* and *Planctomycetes* were detected only with monensin-treated wastewater (Table 3, Figure 4).

Sequences of *Proteobacteria,* nearly non-detectable in clone libraries from wastewater, increased to 46% in monensin-treated wastewater, whereas sequences of *Firmicutes* decreased from 93% to 46% with monensin treatment (Table 3). Although proteobacteria dominated the top 20 sequences with monensin, we did not identify any sequences for the proteobacteria species *E. coli* in clone libraries from wastewater treated with or without monensin.

### Effect of Monensin on Protozoa and EcO157 in Sonneborn Medium

**Figure 5:**
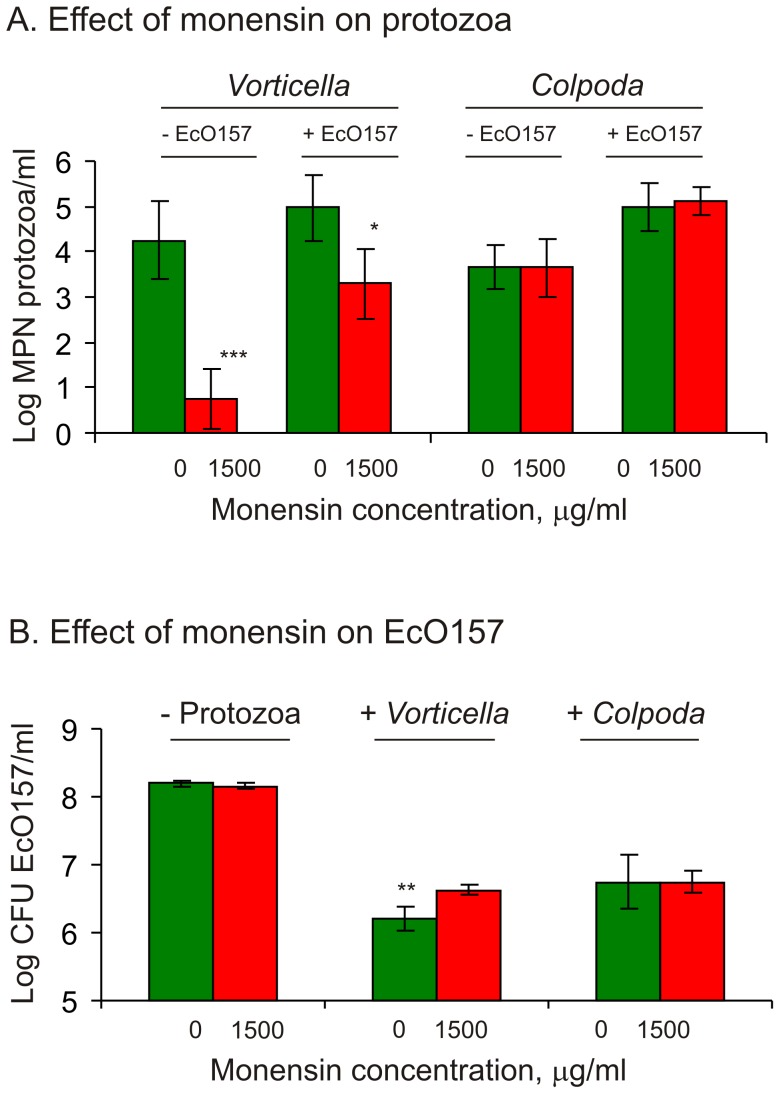
Effect of monensin on the growth of EcO157 and protozoa isolated from dairy wastewater. Changes in protozoan numbers (A) in response to supplementation of monensin or EcO157 and changes in EcO157 populations (B) in the presence or absence of protozoa or monensin were monitored after a 6-day incubation in PBS supplemented with 10% Sonneborn medium. Values are averages of triplicates. Bonferroni post tests: * = *P*<0.05; ** = *P*<0.01; and *** = *P*<0.001.

Since most protozoa characterized from dairy wastewater could not be isolated in pure cultures, tests to confirm the effect of monensin were conducted with two protozoa (*V. microstoma* and *C. aspera*) isolated previously from wastewater from the same lagoon. Numbers of *Vorticella* decreased significantly (*P = *0.0003, F_monensin_ = 35.9), but the populations of *Colpoda* were unaffected (*P = *0.879) in the presence of 1500 µg/ml monensin during a 6-day incubation in reduced strength Sonneborn medium (Figure 5A). The inhibitory effect of monensin was noticed both in the presence or absence of EcO157 as a supplementary food source. Vorticellids decreased from 4.3±0.9 to 0.8±0.7 log MPN in EcO157-free monensin-supplemented media. Although, both protozoa significantly (*P*<0.0001, F = 158) consumed EcO157 cells with or without monensin, vorticellids consumed significantly more (*P*<0.01, t = 5.4) EcO157 cells in monensin-free media (Figure 5B), whereas colpodids consumed EcO157 cells equally well (*P = *0.784) in the presence or absence of monensin. Thus, monensin supplemented EcO157-*Vorticella* treatments contained 2.6 times more EcO157 cells and resulted in a significant increase in D-value for EcO157 from 3.6±0.4 d to 4.8±0.3 days. EcO157 populations in the absence of protozoa were unaffected by monensin treatment (*P*>0.05, t = 0.45; Figure 5B).

## Discussion

Monensin, a dietary feed supplement, extended survival of pathogenic EcO157 in dairy wastewater by inhibiting protozoa that consume EcO157 and by eliminating gram-positive bacteria that compete for nutrients. In addition, we have shown that the antibiotic is not inhibitory to EcO157 and gram-negative bacteria and facilitated the growth of members of *Proteobacteria* undetectable from monensin-free wastewater.

EcO157 survived appreciably longer in monensin-treated wastewater as indicated by >6-fold increase in D-value compared to untreated water. Previous studies also documented resistance of EcO157 [26] and other gram-negative bacteria [24], [25] to monensin under *in vitro* conditions. Since monensin did not stimulate the growth of EcO157 directly (Figure 5B), we reasoned that the extended survival of EcO157 with monensin corresponded to a significant decrease in specific predatory protozoa in wastewater. A 60% decrease in ciliate sequences (Table 2) corresponded to elimination of all colpodean and oligohymenophorean ciliates, and a significant decrease of *Oxytricha* (Figure 3), all of which are consistent with observed increases in EcO157 numbers with monensin treatment. Consistent with the elimination of *V. microstoma* in monensin-treated wastewater (Figure 3), pure culture studies confirmed inhibition of verticellids (Figure 5A) coupled with significant increases of EcO157 (Figure 5B). Similarly, monensin decreased ciliates that resulted in increases in *E. coli* populations in rumen fluids [25].

Protozoa characterized from wastewater appear not to originate from dairy animals, as evidenced by our lack of detection of any of the rumen ciliates (*Entodinium*, *Epidinium, Dasytricha, Ophryoscolex, Ostracodinium, Diplodinium,* and *Metadinium*) in wastewater [23]. It is noteworthy that these protozoa were undetectable in wastewater even though they constitute half of the microbial biomass in rumen [9] and are released into lagoons as manure washings. The failure to survive outside the rumen might be attributed to the absence of anaerobic conditions unique to rumen ecosystems amenable to maintain protozoa. Many of the well characterized protozoa are from aquatic habitats [37], but *Vorticella* and *Oxytricha* are known indicators of water quality in aerobic [38] or anaerobic [39] wastewater treatment systems.

Protozoan community structure was altered by monensin by nearly eliminating all colpodean ciliates while significantly increasing the growth of biflagellate bicosoecids, cercozoans and a spirotrichean ciliate *Cyrtohymena citrina* (Table 2, Figure 3). Since these represented 92% of all sequences (Figure 3), whereas, untreated wastewater contained *Cyrtohymena* at 1% of the total sequences, it seems worthwhile to investigate the role of *Cyrtohymena*, in addition to cercozoans and flagellates, in extending the survival of EcO157 by serving as alternate hosts [40]–[42]. However, shielding EcO157 from predators while supplying nutrients from wastewater extended the survival of EcO157 with a 90% decline rate increasing to 18 days [6]. The increased proliferation of minor members of the protozoan communities (Figure 3) with monensin may result from increased availability of food due to elimination of the most predominant protozoa that compete for food sources. In addition, the enriched protozoa might be resistant to monensin as indicated by the absence of inhibition of *C. aspera* in media supplemented with monensin (Figure 5A). *C. aspera* was detected by 18S rRNA gene sequencing only in wastewater treated with monensin (Figure 3). Predictably, *V. microstoma,* a monensin-sensitive organism that was eliminated from wastewater (Figure 3) also declined in numbers rapidly from media containing monensin (Figure 5A). To our knowledge, this is a first report on protozoan community shifts caused by monensin in wastewater.

Monensin similarly altered bacterial communities in wastewater from almost all gram-positives to a high proportion of gram-negatives and reduced the species diversity. Although no other data exist on the impact of monensin in the environment, bacterial community shifts leading to increases in populations of gram-negatives in the rumen of lactating dairy cows were reported [43] and the altered communities did not revert back to the original state after monensin withdrawal from the feed [44]. Thus, considering that several gram-negative pathogens associated with contaminated fruits and vegetables causing outbreaks can originate from dairy and feedlot environments, it seems worthwhile to monitor whether such community shifts are wide-spread in these same environments.

Members of *Firmicutes* represented 93% of all sequences characterized from wastewater and 100% of the 20 predominant organisms were gram-positive. Similarly, firmicutes predominated in waters from the lagoon and manure separator pit from a dairy in the San Joaquin Valley of California; gram-positives accounted for 80 to 90% of all sequences [45]. The decrease in gram-positive bacteria from 98% to 33% of the top 20 sequences with monensin is predictable as the ionophore specifically targets gram-positives [9], [46], [47], causing a 50% decrease in growth of ruminal gram-positive bacteria within 48 h at a concentration as low as 0.3 µg/ml [46]. Gram-positives are extremely sensitive and even the environmentally persistent *Mycobacterium avium paratuberculosis* that survives for >2 years in soils [48] was inhibited by monensin at 0.4 µg/ml, a result leading to a proposal to use the compound to prevent bovine paratuberculosis [49]. *Clostridia* are particularly sensitive to monensin as they decreased from 83% to 24% of the top 20 sequences in monensin-treated wastewater. In addition, members of genus *Clostridium* produce more extracellular protein toxins than any other bacterial genera [50] and *Clostridium butyricum* in particular has been reported to inhibit the growth and shiga-toxin production of EcO157 [51]. Thus, the decreases in clostridia by monensin may have also aided in increased survival of EcO157 in dairy wastewater.

The increase in proteobacteria from 0 to 62% of clones from the top 20 sequences characterized from monensin treated wastewater, without decreasing EcO157 populations even at a high concentration of 1500 µg/ml in a nutrient medium, confirms the resistance of gram-negatives to the antibiotic and, in particular, pathogenic EcO157. The concentration of monensin used in this study was high considering that it is soluble at concentrations in water (pH 7) no higher than 4.8 µg/ml (http://msds.xh1.lilly.com/Rumensin%20Premix.pdf). This was necessary to detect any evidence of the extended survival of EcO157, considering that monensin degrades rapidly in soil with a half-life of only about 4 days [31]. In one study, the minimum inhibitory concentration exceeded 2048 µg/ml for clinical isolates of *E. coli* O157 and other strains carrying the shiga-toxin gene *stx_2_* [30]. Coincidentally, a higher prevalence of shiga-toxin positive EcO157 was observed in herds using dietary monensin as a supplement [52] and *E. coli* populations increased 50-fold even at a low concentration of monensin at 5.8 µg/ml in artificial rumen [25]. Thus, facilitating the growth of *E. coli* and other gram-negative bacteria [46], while suppressing gram-positives, was exploited for developing monensin-based media for selective enumeration of *E. coli* and gram-negative bacteria from food and environmental samples [53]. Furthermore, half of the cattle-fed monensin was excreted as the parent molecule [54] and detected at 3.2 µg/ml in run-off water from manure stockpiles [55], in dairy lagoons and in shallow ground water samples from dairy production areas [56]. Therefore, it is important to measure the true increases in gram-negative bacteria along with an extended survival of EcO157 in agricultural environments.

The ability of gram-negatives to grow in the presence of monensin was further supported by a 2 log increase in total aerobic bacteria, maintaining an elevated level of >8 log CFU/ml throughout the incubations in monensin-treated wastewater (Figure 1). The increase in gram-negative bacterial sequences from 1 to 46% (Table 3) with monensin further confirms that the increase in aerobic bacteria represented gram-negatives predominantly. Furthermore, monensin applied 4 days before the zero-day bacterial count might have already inhibited gram-positives, thus making available more nutrients for gram-negative bacterial growth.

An unintended consequence of using monensin to inhibit protozoa resulted in enrichment of an uncultured *Advenella* species known to degrade a chloro-*s*-triazine herbicide, terbuthylazine, when present in ground water [57]. The enrichment of *Advenella,* representing one-third of all characterized sequences, indicated that this organism likely used monensin for growth. There are no known reports of microbial consumption of monensin, rather, the compound is known to degrade rapidly in soil [31].

The observed community shifts favoring gram-negative bacteria coupled with extended survival of EcO157 by monensin in wastewater support the earlier findings of resistance of EcO157 [30] and higher incidence of EcO157 in herds treated with dietary monensin [52]. More importantly, the identification of EcO157 as a foodborne pathogen coinciding with the large scale use of ionophores as feed supplements raises concerns that ionophores enhanced the ability of EcO157 to become established as intestinal flora of cattle. Although no evidence exists that exposure to monensin causes EcO157 to acquire cross-resistance to antibiotics used in human medicine, prudent use of ionophores is warranted as monensin treatment resulted in extended survival of monensin-resistant EcO157 populations by eliminating predatory protozoa. A recent publication [58] reported similar community shifts favoring proteobacteria along with increases in *E. coli* populations in the intestinal microbiome of swine fed with diet supplemented with performance-enhancing antibiotics. Since >90% of agricultural antibiotics are used for growth promotion [59], the European Union restricted their use for therapeutic purposes only [60] and the US FDA issued guidance recommending the judicious use of antibiotics for food producing animals [61].

In summary, these results reflect the complexity of the ecology of the dairy and wastewater environments and the dynamic fluctuations that could occur as a result of an imbalance in microflora, pathogen, protozoa, and antimicrobial compounds as feed supplements, as examples. We observed significant decreases in pathogenic EcO157 populations due to resident protozoa present in wastewater, but survival was extended significantly by inhibiting the predatory protozoa with monensin. Although one must be cautious about the protective nature of some protozoa in extending the survival and proliferation of pathogenic bacteria, protozoa may be a beneficial on-site strategy to minimize enteric pathogen contamination of minimally processed ready-to-eat fruits and vegetables. These observations may assist in explaining the differences in pathogen incidence in some environments [15], [42], but further work with different strains and microcosms from dairies that restrict growth-promoting ionophores from cattle diet (organic dairies) will be required to determine if these results indicate a general phenomena that can be exploited as a strategy for controlling pathogens in produce production environments.

## Supporting Information

Figure S1 **Survival of EcO157 in dairy wastewater treated with or without monensin.** EcO157 populations (Panel A) in each replicate (red; ◊, ▵, □) of monensin-treated wastewater were plotted separately to show replicate differences in D-values (◊, 2.6 d; ▵, 2.9 d; and □, 5.1 d). D-value for EcO157 in untreated wastewater was 0.8±0.0 d (green •; average of triplicates). D-values were based on EcO157 populations remaining after 6 days. Compared to monensin treatment, EcO157 numbers decreased significantly in untreated wastewater (*P*<0.0001, t_1–6days_ = 6.2–21.2, F_time_ _intervals_ = 384). Corresponding data on native bacteria and protozoa are shown in panel B. Two-way repeated-measures ANOVA was used to determine the significance of monensin-treatment on bacterial and protozoan populations during a 6-day period when EcO157 was not detected in untreated water. Bonferroni post-hoc t-tests: * = *P*<0.05 and **** = *P*<0.0001.
(TIF)
