# Supplementary figures and images for: Altered Protozoan and Bacterial Communities and Survival of Escherichia coli O157:H7 in Monensin-Treated Wastewater from a Dairy Lagoon
Source: PLoS One. 2013 Jan 22;8(1):e54782. doi: 10.1371/journal.pone.0054782 (PMC3551901; doi:10.1371/journal.pone.0054782)

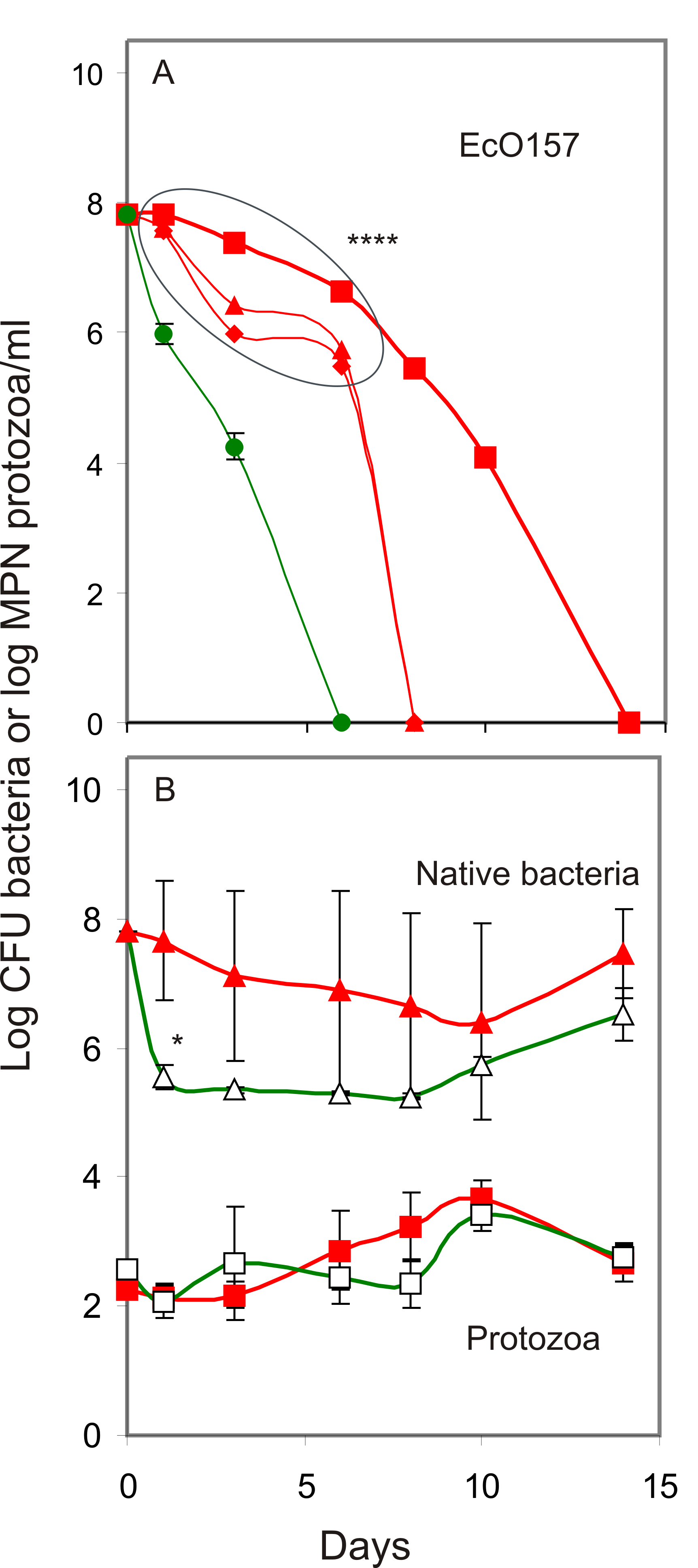

Supplement: Figure S1 — Survival of EcO157 in dairy wastewater treated with or without monensin. EcO157 populations (Panel A) in each replicate (red; ◊, ▵, □) of monensin-treated wastewater were plotted separately to show replicate differences in D-values (◊, 2.6 d; ▵, 2.9 d; and □, 5.1 d). D-value for EcO157 in untreated wastewater was 0.8±0.0 d (green •; average of triplicates). D-values were based on EcO157 populations remaining after 6 days. Compared to monensin treatment, EcO157 numbers decreased significantly in untreated wastewater (P<0.0001, t1–6days = 6.2–21.2, Ftime intervals = 384). Corresponding data on native bacteria and protozoa are shown in panel B. Two-way repeated-measures ANOVA was used to determine the significance of monensin-treatment on bacterial and protozoan populations during a 6-day period when EcO157 was not detected in untreated water. Bonferroni post-hoc t-tests: * = P<0.05 and **** = P<0.0001. (TIF) [file pone.0054782.s001.tif]
